# Nefropatía *full house* no lúpica en pediatría: reporte de dos casos

**DOI:** 10.7705/biomedica.4863

**Published:** 2020-06-30

**Authors:** Gustavo Adolfo Guerrero, Luis Francisco Guerrero, Tatiana González

**Affiliations:** 1 Servicio de Nefrología Pediátrica, Hospital Infantil Napoleón Franco Pareja, Cartagena, Colombia Hospital Infantil Napoleón Franco Pareja Cartagena Colombia; 2 Departamento de Pediatría, Universidad de Cartagena, Cartagena, Colombia Universidad de Cartagena Departamento de Pediatría Universidad de Cartagena Cartagena Colombia; 3 Servicio de Reumatología Pediátrica, Hospital Infantil Napoleón Franco Pareja, Cartagena, Colombia Hospital Infantil Napoleón Franco Pareja Cartagena Colombia

**Keywords:** nefritis lúpica, lupus eritematoso sistémico, enfermedades renales, Lupus nephritis, lupus erythematosus, systemic, kidney diseases

## Abstract

La nefropatía *full house* se refiere a la detección simultánea de depósitos de IgA, IgG, IgM, C3 y C1q en la inmunofluorescencia, lo que generalmente indica la presencia de nefritis lúpica. Hay pacientes con este patrón de inmunofluorescencia, pero con serología negativa para autoanticuerpos, por lo que no se les puede diagnosticar un lupus eritematoso sistémico. Este tipo de nefropatía, en la que no se presentan otros criterios para lupus, se denomina nefropatía *full house* no lúpica.

En esta presentación, se describen dos casos: un paciente que ingresó con una glomerulonefritis rápidamente progresiva y una paciente con síndrome nefrótico, ambos con serología negativa para autoanticuerpos, hallazgos en la biopsia renal indicativos de nefritis lúpica de clase IV y un patrón *full house* en la inmunofluorescencia. La nefropatía *full house* no lúpica tiene rasgos histológicos similares a los de la nefritis lúpica y, probablemente, sus bases fisiopatológicas son parecidas. Sin embargo, se necesitan estudios prospectivos para conocer los factores de riesgo y el pronóstico renal, y poder hacer sugerencias sobre tratamientos específicos.

## Abstract (en)

Full house nephropathy is defined as the simultaneous detection of IgA, IgG, IgM, C3, and C1q deposits by immunofluorescence, usually indicating lupus nephritis. There are patients with this immunofluorescence pattern, but with negative autoantibody serology, which means they cannot be diagnosed with systemic lupus erythematosus. Patients presenting with full house nephropathy but no other criteria for lupus are diagnosed as having nonlupus full house nephropathy.

Here, we describe two cases: A male patient who debuted with rapidly progressive glomerulonephritis and a female patient with nephrotic syndrome. Both had negative autoantibody serology, findings in the renal biopsy of class IV lupus nephritis and a full house immunofluorescence pattern. Histological findings in non-lupus full house nephropathy are similar to those in lupus nephritis and, probably, similar physiopathological bases. However, prospective studies are needed to determine risk factors and the renal prognosis and to make suggestions for specific treatments.

En las biopsias renales de los pacientes con lupus eritematoso sistémico, se puede encontrar un patrón de inmunofluorescencia denominado nefropatía *full house*, la cual se refiere a la detección simultánea de depósitos de IgA, IgG, IgM, C3 y C1q ^1^^,^^2^. En pacientes con signos y síntomas extrarrenales de lupus eritematoso sistémico, con nefropatía *full house* en la biopsia renal y con autoanticuerpos positivos, se hace diagnóstico de nefritis lúpica.

Según los nuevos criterios de clasificación del lupus eritematoso (*The Systemic Lupus International Collaborating Clinics,* SLICC)*,* con la única manifestación renal probada por biopsia en presencia de autoanticuerpos puede hacerse el diagnóstico de nefritis lúpica ^3^. Sin embargo, existen casos que presentan lesiones histopatológicas con patrón de inmunofluorescencia *full house* sin hallazgos serológicos de autoanticuerpos y sin otros signos ni síntomas extrarrenales de lupus eritematoso, por lo que dicho diagnóstico no puede hacerse ^4^

En la literatura especializada se encuentran algunos informes de casos de pacientes con nefritis *full house* que en el transcurso de la enfermedad desarrollan criterios diagnósticos de lupus eritematoso ^5^. Los hallazgos histológicos de pacientes con nefropatía *full house* y ninguna otra manifestación de lupus se han denominado como nefropatía *full house* no lúpica ^4^.

Wen, *et al.*^4^, fueron los primeros en describir un grupo de 24 pacientes con nefropatía *full house* no lúpica y establecer el espectro clínico-patológico de la enfermedad. Existe un grupo de enfermedades bien establecidas que pueden manifestarse como nefropatía *full house* no lúpica, por ejemplo, diabetes mellitus, hepatopatías, glomerulopatías primarias e infecciones ^4^^,^^6^^-^^9^.

Se reportan dos pacientes pediátricos con nefropatía *full house* sin hallazgos serológicos asociados con lupus eritematoso, diagnosticados en un hospital pediátrico de Cartagena de Indias, Colombia.

## Caso clínico 1

Se trata de un paciente de 10 años de edad, de sexo masculino, con cuadro clínico de dos semanas de evolución consistente en edema progresivo que afectaba la región facial y las extremidades.

Una semana antes del ingreso, había presentado malestar general, hematuria macroscópica y fiebre, por lo cual fue llevado a consulta a un centro hospitalario de segundo nivel, donde se le detectó el edema de cara y extremidades inferiores, y presión arterial alta, por lo que fue hospitalizado. El paciente había presentado faringoamigdalitis un mes antes, sin otros antecedentes de importancia. Se inició el tratamiento antihipertensivo y, debido a la elevación progresiva de azoados, se le remitió a un hospital de tercer nivel de atención.

En el examen físico se registró un peso de 34,5 kg (z=+0,03), su talla era de 150 cm (z=+1,20), la tensión arterial controlada era de 100/70 mm Hg, y el examen físico fue normal, con excepción del leve edema facial.

En el cuadro 1 se presentan los resultados de los exámenes paraclínicos a su ingreso. Además, la detección de anticuerpos IgM e IgG para citomegalovirus, toxoplasma y virus de Epstein-Barr fue negativa. Los títulos de los anticuerpos antinucleares (ANA), anti-ADN nativo, anti- lupus, anticuerpos anti-Ro, anticuerpos anti-RNP y anticuerpos anti-SM, fueron negativos, así como los anticuerpos anticardiolipina IgG e IgM y el anticoagulante lúpico a los 3,8 segundos (control: 34,2 segundos). En la ecografía renal y de vías urinarias, los riñones aparecían de tamaño normal con aumento difuso en la ecogenicidad; en el ecocardiograma, no se registraron alteraciones y la fracción de eyección fue de 77 %.

**Cuadro 1:** Estudios iniciales de laboratorio

	Caso 1	Caso 2
Hemograma			
Hemoglobina (g/dl)	7,7	15
Hematocrito (%)	22	45
Leucocitos (por mm^3^)	8.220	12.500
Neutrófilos (%)	57	41
Linfocitos (%)	27	52
Monocitos (%)	12	7
Plaquetas (por mm^3^)	325.000	410.000
Pruebas renales			
Nitrógeno ureico (mg/dl)	71	5,3
Creatinina (mg/dl)	5	0,85
Uroanálisis			
pH	5	6
Densidad	1,025	1,02
Proteínas (mg/dl)	100	300
Hemoglobina	++	++
Leucocitos (por campo)	8-10	5-10
Hematíes (por campo)	Campos llenos	Campos llenos
Proteinuria en 24 horas (mg/24 horas)	5.469	70.801
(mg/m^2^/hora)	190	1.891
Índice proteinuria/creatinuria	12,72	78,72
Urocultivo	Negativo	Negativo
Electrolitos séricos			
Sodio (mEq/L)	124	135
Potasio (mEq/L)	4,1	3,9
Cloro (mEq/L)	106	103
Calcio (mg/dl)	5,5	6,8
Magnesio (mg/dl)	1,2	1,8
Fósforo (mg/dl)	10,9	3,4
Metabólico			
Colesterol total (mg/dl)	250	349
HDL (mg/dl)	20	94
LDL (mg/dl)	200	222,6
Triglicéridos (mg/dl)	343	162,4
Albúmina (gr/dl)	1,8	2,1
Complemento sérico			
C3 (VR: 90-180 mg/dl)	52,4	103,2
C4 (VR: 10-40 mg/dl)	12,5	43,9

VR: valor de referencia

Durante la hospitalización, se evidenció un incremento progresivo y rápido de los azoados, lo que llevó a suponer la presencia de glomerulonefritis de progresión rápida por impresión clínica. Se le practicó una biopsia renal y con microscopía de luz se evidenciaron 15 glomérulos, en 13 de los cuales se observó una grave y extensa proliferación extracapilar con formación de semilunas celulares, atrofia tubular en el 25 %, cilindros hialinos y hemáticos, intersticio con infiltrado linfocitario difuso y fibrosis presente en el 25 %; las coloraciones especiales de ácido periódico de Schiff (*periodic acid-Schiff*, PAS), tricrómico y plata, evidenciaron los cambios de cronicidad. Con la inmunofluorescencia se observó un patrón *full house*.

En la microscopía electrónica, se evidenciaron dos glomérulos y se registraron depósitos intramembranosos del tipo de inmunocomplejos, pérdida de pedicelos hasta en el 80 %, presencia de proliferación celular que ocupaba el espacio de Bowman, depósitos subendoteliales frecuentes y electrodensos del tipo de inmunocomplejos, y expansión mesangial con depósitos electrodensos del tipo de inmunocomplejos. El índice de actividad fue de 19/24 y el de la cronicidad fue de 5/12. Con base en estos resultados, se concluyó que el paciente presentaba glomerulonefritis de progresión rápida mediada por inmunocomplejos sugestiva de una nefritis lúpica de clase IV, actividad global grave y cronicidad extensa.

El paciente evolucionó con valores de presión superiores al percentil 99, asociados con oliguria, y fue tratado con varios antihipertensivos y hemodiálisis por incremento marcado de azoados e hipercalemia. Se le administraron tres pulsos diarios de metilprednisolona y dos de ciclofosfamida con un intervalo de un mes. Los especialistas en nefrología y reumatología pediátricas decidieron continuar el tratamiento inmunosupresor oral con micofenolato de mofetilo y un corticoide (deflazacort).

Al año de seguimiento, el paciente no había tenido recaídas, su tensión era normal, su función renal estaba estable sin necesidad de diálisis. Se encontraron niveles de creatinina de 1,3 mg/dl, nitrógeno ureico de 14,9 mg/dl, C3 de 100 mg/dl, C4 de 26 mg/dl, ANA, anti-DNA y anticuerpos frente al citoplasma de neutrófilos (ANCA) negativos, una tasa de filtración glomerular (Schwartz) de 58 ml/minuto/1,73, y proteinuria subnefrótica. El paciente continuó en tratamiento con 15 mg de deflazacort día de por medio, 500 mg de micofenolato de mofetilo cada 12 horas, y clonidina y losartán para la hipertensión.

## Caso clínico 2

Se trata de una paciente de 13 años de edad, de sexo femenino, con un cuadro clínico de un mes de evolución consistente en edema que se inició en los miembros inferiores. Fue llevada al centro de salud donde se la trató ambulatoriamente por celulitis, con antibióticos no especificados. El edema progresó hasta afectar su rostro, y presentó orina espumosa y oliguria. No presentaba antecedentes clínicos de importancia.

En el examen físico, su peso fue de 54,5 kg (z=+0,58), la talla de 161 cm (z=+0,20), y su tensión arterial de 120/80 mm Hg. El examen físico fue normal, excepto por el leve edema facial y el de grado II en los miembros inferiores.

Los resultados de los exámenes paraclínicos de ingreso se presentan en el cuadro 1. Se encontró proteinuria en rango nefrótico, además de dislipidemia e hipoalbuminemia, pero no había afectación ganglionar ni articular, como tampoco dolor.

Teniendo en cuenta factores de riesgo como la edad y sexo, en el diagnóstico diferencial se decidió descartar una colagenopatía, por lo que se solicitaron exámenes para detectar los niveles de anticuerpos y una biopsia renal.

Los anticuerpos contra hepatitis C y B y HIV fueron negativos; la IgG para rubéola fue de 12,5 UI/ml y la IgM de 0,9 UI/ml; para herpes I, la IgG fue de 25,48 UI/ml y la IgM de 3,7 UI/ml; para herpes II, la IgG fue de 13,5 UI/ml y la IgM de 2,3 UI/ml; el anti-ADN, el ANA y el ANCA no fueron reactivos, y en la ecografía renal y de vías urinarias se encontraron riñones de tamaño normal, con aumento difuso de la ecogenicidad y pérdida de la diferenciación corticomedular.

Se practicó una biopsia renal y, con microscopía óptica, se registraron 12 glomérulos, dos con esclerosis global y uno con esclerosis segmentaria, así como nefritis intersticial del 15 al 30 % e infiltrado mononuclear con presencia focal de eosinófilos. Se encontró: vacuolización tubular (++), proteínas de reabsorción, fibrosis intersticial del 10 % (puntaje de cronicidad: 1) y atrofia tubular menor de 5 % (puntaje de cronicidad: 0) sin vasculitis.

En la microscopía electrónica, se evaluaron dos glomérulos, uno con evidencia de esclerosis segmentaria, retracción de la membrana basal, doble contorno e interposición mesangial asociado con depósitos electrodensos con morfología de complejos inmunitarios de localización subendotelial e intramembranosos, así como mesangio con depósito de complejos inmunitarios, sin proliferación celular ni otros depósitos.

Se concluyó que la paciente presentaba una nefropatía mediada por complejos inmunitarios, de patrón membranoproliferativo y hallazgos indicativos de nefritis lúpica de clase IV. Los especialistas en nefrología y reumatología pediátricas consideraron que cursaba con un síndrome nefrótico secundario a nefropatía *full house* no lúpica, sin compromiso de ningún otro sistema orgánico, excepto el renal, y sin autoanticuerpos.

Se inició tratamiento con prednisolona con mejoría clínica y disminución del edema. La paciente tuvo una recaída con proteinuria de 41.910 mg/24 horas y se le administraron tres pulsos de metilprednisolona. Durante el seguimiento, persistió el edema de miembros inferiores con hipoalbuminemia y proteinuria en rango nefrótico, por lo que se le administró un pulso de ciclofosfamida al mes durante seis meses, con lo cual se obtuvo mejoría parcial, con evolución favorable de los edemas y albúmina normal; no obstante, la proteinuria persistió en rango nefrótico, así como la hipercolesterolemia, por lo que se inició tratamiento con micofenolato mofetilo.

Al año de seguimiento, la paciente tenía tensión normal, no había presentado recaídas, la proteinuria era subnefrótica y conservaba la función renal.

### Consideraciones éticas

Se siguieron las normas éticas para la investigación en seres humanos contenidas en la Resolución 008430 de 1993 del Ministerio de Salud de Colombia y se mantuvo la confidencialidad de la información de los pacientes.

## Discusión

Se reportan dos casos: un niño con glomerulonefritis de progresión rápida y con antecedente de infección, a quien se le practicó una biopsia renal que evidenció un patrón histológico concordante con glomerulonefritis proliferativa difusa y semilunas celulares en más del 50 %, y una niña con síndrome nefrótico y biopsia indicativa de nefritis lúpica de clase IV. Los dos pacientes presentaban un patrón *full house* de inmunofluorescencia. Los casos se diagnosticaron como nefropatía *full house* no lúpica y en ambos se administró tratamiento de inmunosupresión.

El término de nefropatía *full house* no lúpica se ha utilizado para describir a un grupo de pacientes que presentan un patrón de inmunofluorescencia *full house* con serología negativa para autoanticuerpos y sin ningún otro criterio de lupus ^4^.

Wen, *et al.*, describieron una serie de 24 pacientes con diagnóstico histopatológico de nefropatía membranosa (46 %), nefropatía por IgA (21 %), glomerulonefritis membranoproliferativa (12,5 %), glomerulonefritis posinfecciosa (12,5 %), nefropatía C1q (4 %) y mesangial no clasificable (4 %). Después de un seguimiento promedio de 24 meses, solo dos pacientes desarrollaron lupus eritematoso ^4^. Por su parte, Martín, *et al.,* reportaron su experiencia en dos centros hospitalarios de Medellín (Colombia) y describieron la historia clínica de 20 pacientes,12 adultos y 8 niños. Durante el seguimiento de 40,9 ± 24,8 meses, dos pacientes presentaron anticuerpos antinucleares, por lo que los autores concluyeron que en el seguimiento siempre se debe estar atento al desarrollo de lupus eritematoso ^10^.

Rijnink, *et al.*, informaron 32 pacientes con nefropatía *full house* no lúpica y un seguimiento promedio de 20 años, durante el cual ninguno desarrolló lupus eritematoso. De estos pacientes, 20 tenían nefropatía *full house* no lúpica idiopática y, 12, nefropatía *full house* no lúpica secundaria. Los primeros presentaron menos hematuria, más proteinuria en rango nefrótico y menos consumo del complemento en la vía clásica que aquellos con lupus. Además, mediante un análisis de regresión multivariable, los autores encontraron que los afectados por la nefropatía *full house* no lúpica con patrón de lesión proliferativa progresaron más rápidamente a enfermedad renal terminal que los pacientes con lupus ^11^.

La combinación de una fisiopatología similar y el mal pronóstico renal de algunos pacientes con nefropatía *full house* no lúpica ^4^^,^^7^^,^^9^^,^^11^, lleva a pensar en la posibilidad de que tengan formas graves de otras enfermedades caracterizadas por una importante activación del sistema inmunológico.

En uno de los casos aquí reportados, el paciente había sufrido faringoamigdalitis un mes antes de presentar el cuadro clínico, por lo cual se consideró la glomerulonefritis posinfecciosa entre los diagnósticos diferenciales y, con base en los estudios practicados, no se descartó como posible desencadenante de la glomerulonefritis de progresión rápida mediada por una enfermedad de complejos inmunitarios. Dos de los 32 pacientes reportados por Rijnink, *et al.*, presentaban glomerulonefritis relacionada con una infección ^11^. En la serie de Wen, *et al.*, el diagnostico final de tres de los pacientes fue de glomerulonefritis posinfecciosa ^4^.

En el estudio retrospectivo más grande de pacientes pediátricos publicado hasta el momento, Ruggiero, *et al.*, reportaron 42 niños y adolescentes en cinco centros de nefrología de Italia describiendo los esquemas terapéuticos utilizados y los resultados obtenidos. Los autores encontraron que, en la inducción, las niñas recibieron una dosis acumulativa de ciclofosfamida más alta que los niños. Además, los niños en etapa prepuberal recibieron dosis de esteroides diarios más altas que los demás púberes. Durante la fase de mantenimiento, no hubo diferencias entre niños y niñas. A los 10 años, la probabilidad de desarrollar enfermedad renal crónica fue de 4,8 % y las recurrencias se dieron solamente en los primeros cuatro años después del diagnóstico, lo que permitió la suspensión progresiva de la inmunosupresión en aquellos pacientes que lograron la remisión ^12^.

En el primer caso aquí reportado, se eligió la opción del tratamiento inmunosupresor por el deterioro progresivo de la función renal, con un esquema de corticoides y ciclofosfamida (2 pulsos) y luego micofenolato de mofetilo oral. El tratamiento de la segunda paciente se inició con esteroides y, debido a la resistencia observada, se optó por la administración de ciclofosfamida endovenosa mensual, que solo después de seis pulsos logró una mejoría parcial, a partir de lo cual se inició el tratamiento con micofenolato de mofetilo.

Los estudios evidencian que son pocos los pacientes que presentan serología positiva para lupus eritematoso durante el seguimiento, sin embargo, existe el riesgo. Por ejemplo, en el estudio de Wen, *et al.*, de 24 pacientes, solo dos tuvieron resultado positivo para ANA ^4^; Martín, *et al.*, también reportaron dos pacientes de los 20 analizados ^10^, en tanto que en el estudio de Rijnink, *et al.*, ninguno de los 32 pacientes analizados presentó positividad ^11^. Nuestra hipótesis frente a esta baja seroconversión es que, con el uso de inmunosupresores, se puede inhibir la formación de autoanticuerpos y evitar el desarrollo del lupus eritematoso.

Entre las pruebas diagnósticas que podrían ayudar a diferenciar la nefropatía *full house* no lúpica de la lúpica, están las inclusiones túbulo- reticulares con la microscopía electrónica ^13^ y la presencia de depósitos inmunes en la inmunofluorescencia de piel ^14^. Dichas inclusiones son muy sugestivas de nefritis lúpica y son raras en otras enfermedades renales. Por lo tanto, la combinación de criterios clínicos, la inmunofluorescencia de piel y la biopsia renal con microscopía electrónica puede ayudar a determinar las formas que evolucionan a lupus eritematoso sistémico ^12^.

No hay estudios que establezcan el tratamiento apropiado para la nefropatía *full house* no lúpica ni recomendaciones para el manejo de estos pacientes. Sin embargo, muchos médicos han notado el riesgo de progresión hacia el lupus, por lo que recomiendan un tratamiento con inmunosupresores similar al propuesto para la nefritis lúpica con lesiones histológicas graves ^7^^,^^15^.

En un estudio observacional, Ruggiero, *et al.*, informaron que los esquemas terapéuticos agresivos de inducción lograron una remisión sostenida en todos los casos de nefropatía *full house*^16^. La opción del tratamiento inmunosupresor agresivo en los pacientes con nefropatía *full house* no lúpica, se basa en el curso progresivo y el mal pronóstico de los casos no tratados. Además, los pacientes tratados que no desarrollan lupus eritematoso sistémico parecen tener un mejor pronóstico que quienes sí lo desarrollan ^12^.

Los pacientes aquí presentados llevan más de un año de seguimiento y su función renal ha sido estable y están en remisión. Además, en el caso 1, se logró suspender la terapia de sustitución renal al mes de iniciado el tratamiento inmunosupresor y, hasta la fecha, la serología para autoanticuerpos sigue siendo negativa en ambos pacientes.

## Conclusión

En la actualidad, no se puede afirmar que la nefropatía *full house* no lúpica haga parte de las manifestaciones histológicas iniciales del lupus eritematoso sistémico ni hay evidencia de que lo sea de alguna otra condición clínica específica. En la nefropatía *full house* no lúpica, los hallazgos histológicos son similares a los de la nefritis lúpica y, probablemente, sus bases fisiopatológicas sean parecidas. Sin embargo, se requieren estudios prospectivos en adultos y niños para determinar los factores de riesgo y los resultados renales para poder recomendar pautas de tratamientos con inmunosupresores específicos.
